# Detection of a new heterozygous pathogenic *NFIA* variant in metopic craniosynostosis with preaxial polysyndactyly: A case report

**DOI:** 10.1016/j.jpra.2025.05.013

**Published:** 2025-06-03

**Authors:** Sipho Simon Nhongo, Eilidh Simpson, Moorthy Halsnad, Meharpal Sangra, Ahad Shafi, Jamie Campbell, Louise Thompson, David Moore

**Affiliations:** a Specialty Registrar, Oral and Maxillofacial Surgery, Scottish National Centre for Craniofacial Surgery, Royal Hospital for Children, Glasgow, UK; b Third Year Medical Student at the University of Glasgow, Glasgow, UK; c Consultant Cranio-Maxillofacial Surgeon, Scottish National Centre for Craniofacial Surgery, Royal Hospital for Children, Glasgow, UK; d Consultant Paediatric Neurosurgeon & Craniofacial Surgeon, Scottish National Centre for Craniofacial Surgery, Royal Hospital for Children, Glasgow, UK; e Consultant in Clinical Genetics, Institute of Genetics and Cancer, Edinburgh, UK; f Clinical Scientist, South East Scotland Genetic Service, Western General Hospital, Edinburgh, UK

**Keywords:** Variant, *NFIA*, Craniosynostosis, Polysyndactyly

## Abstract

Craniosynostosis is a congenital condition caused by the early fusion of one or more skull vault sutures during embryological development, resulting in an abnormal head shape. This condition has been linked to many gene variants. The authors report a case of a novel heterozygous pathogenic *NFIA* variant, in a young boy presenting with metopic craniosynostosis with preaxial polysyndactyly. Craniosynostosis is a rarely reported feature of *Nuclear factor I/A (NFIA)* related disorder. This report highlights the investigations that were performed to identify this variant and details the child’s surgical management and outcome.

## Introduction

Craniosynostosis is a congenital malformation caused by premature fusion of one or more skull vault sutures, resulting in abnormal head shape. Complications arise from compromised intracranial volume and may include raised intracranial pressure and visual impairment. Hearing loss or intellectual disability may be caused by the underlying gene defect or may occur secondarily. The global incidence is approximately 1–1.28 in 2,000 live births. Craniosynostosis can be further categorized into syndromic and non-syndromic types, with syndromic cases in the minority, accounting for 15–30 % of all presentations. Variants in at least 60 genes have been identified as recurrently causing craniosynostosis, and commonly implicated genes include *EFNB1, ERF, FGFR2, FGFR3, SMAD6, TCF12 TWIST1*.^1^

The *Nuclear factor I/A (NFIA*) gene (OMIM*600,727) is located on chromosome 1p31.3 and originates from the nuclear factor I (NFI) family of transcription factors. NFI proteins are crucial in central nervous system (CNS) development and functional faults may result in brain malformations, craniofacial and urinary tract anomalies.^2^^,^^3^ Haploinsufficiency of *NFIA* causes *NFIA*-related disorder (OMIM #613,735, officially named ‘brain malformations with or without urinary tract defects’ or (BRMUTD)) – a syndrome characterized by abnormalities such as macrocephaly, corpus callosum hypoplasia, hydrocephalus or ventriculomegaly, urinary tract defects, dysmorphic features, seizures and developmental delay. Less than thirty patients affected by this disorder have been identified and most arise from de novo variants.^3^

Craniosynostosis is a rarely reported feature of *NFIA*-related disorder, with only six documented cases–three of which were in a single family.^4^, ^5^, ^6^, ^7^

Polydactyly and polysyndactyly are rarely reported features of *NFIA-*related disorders. One article describes a 6-year-old boy who presented with preaxial polydactyly, bifid great toes, corpus callosum hypoplasia and macrocephaly with no craniosynostosis reported.^2^

There have been a few reports of genetic variants causing both polydactyly and craniosynostosis simultaneously, including *FGFR, CRPT1* and *CRPT2*, leading to syndromes such as Apert syndrome and Carpenter syndrome. None of the current literature report a link between an *NFIA* variant resulting in both craniosynostosis and polydactyly.

Here, the authors present a case of an infant boy with metopic craniosynostosis, polysyndactyly and speech delay resulting from a de novo, pathogenic, heterozygous variant in the *NFIA* gene. This variant has not been reported in literature or within the gnomAD database.

## Case report

The proband is a 9-week-old male who was referred to the Scottish National Craniofacial Service at the Royal Hospital for Children, Glasgow. Assessment was requested after parents and clinicians noted an abnormally large and misshapen head at birth.

**Figure 1:**
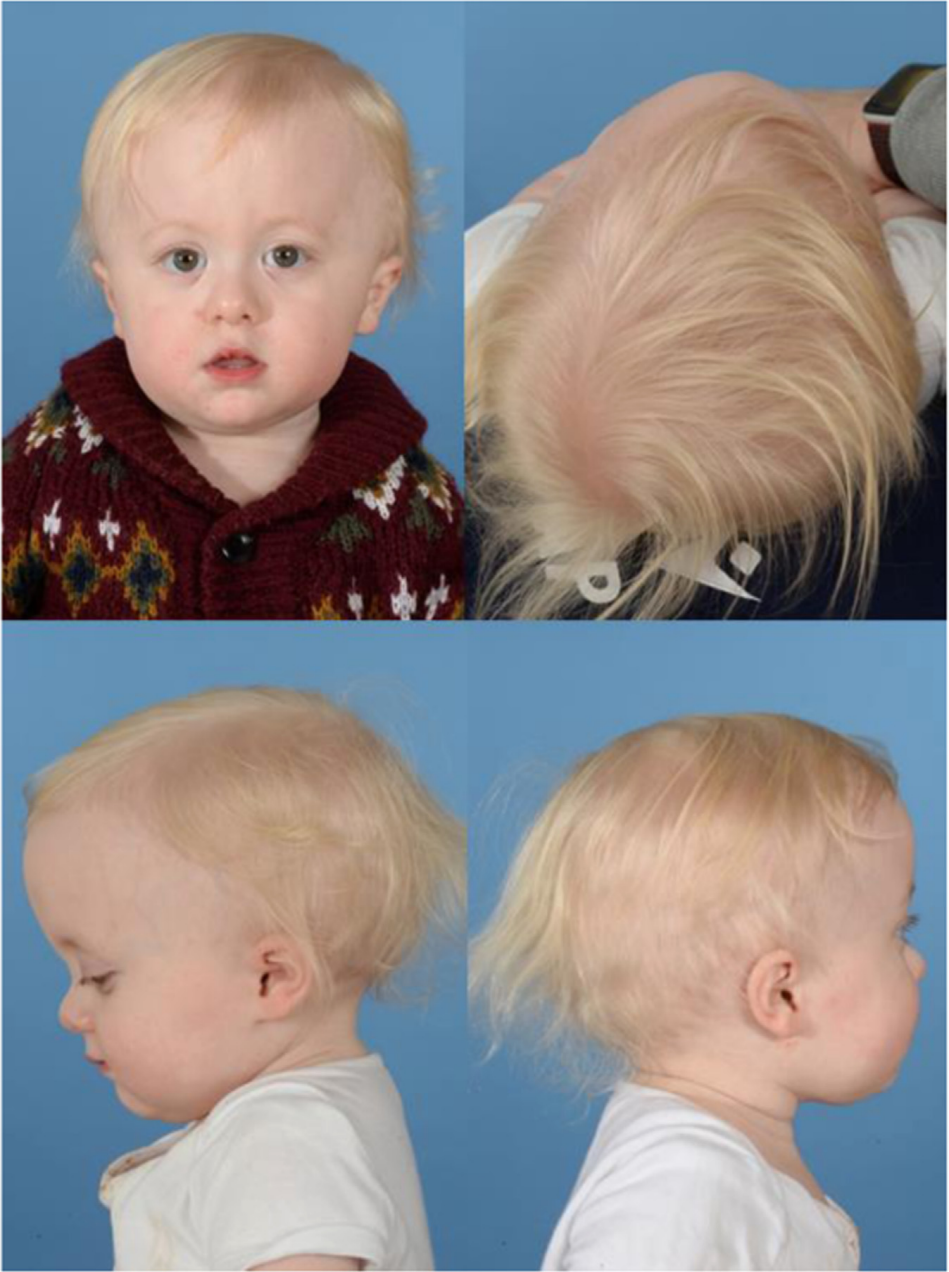
Preoperative clinical photographs at 21 months 19 days of age.

The child was born at term to non-consanguineous parents, via C-section after prolonged induced labor. Birth weight was 3300 g (29th centile, Z −0.56). Clinical inspection at 8 months of age revealed a trigonocephalic appearance with a prominent metopic ridge, bitemporal pinching, hypotelorism and compensatory biparietal widening (See Figure 1). Macrocephaly was also noted, with an occipito-frontal circumference OFC >99th centile (49.5 cm at 8 months old(*Z* + 3.99) and 52 cm at 14 months (*Z* + 4.17)). The anterior fontanelle was open and normotensive.

Ophthalmological examination showed no evidence of raised intracranial pressure.

Limb inspection revealed bilateral great toe complex polysyndactyly (bifid great toes), with duplication of the distal phalanx of each great toe. While both thumbs appeared broad, x-rays showed no upper limb bony abnormalities.

Magnetic resonance imaging (MRI) at 2 months of age reported corpus callosum hypoplasia with dysgenesis. A frontal ridge suggestive of metopic synostosis was noted, however a computed tomography (CT) scan was suggested to better elucidate bony anatomy. Head CT scan was performed at 13 months and confirmed the findings.

**Figure 2:**
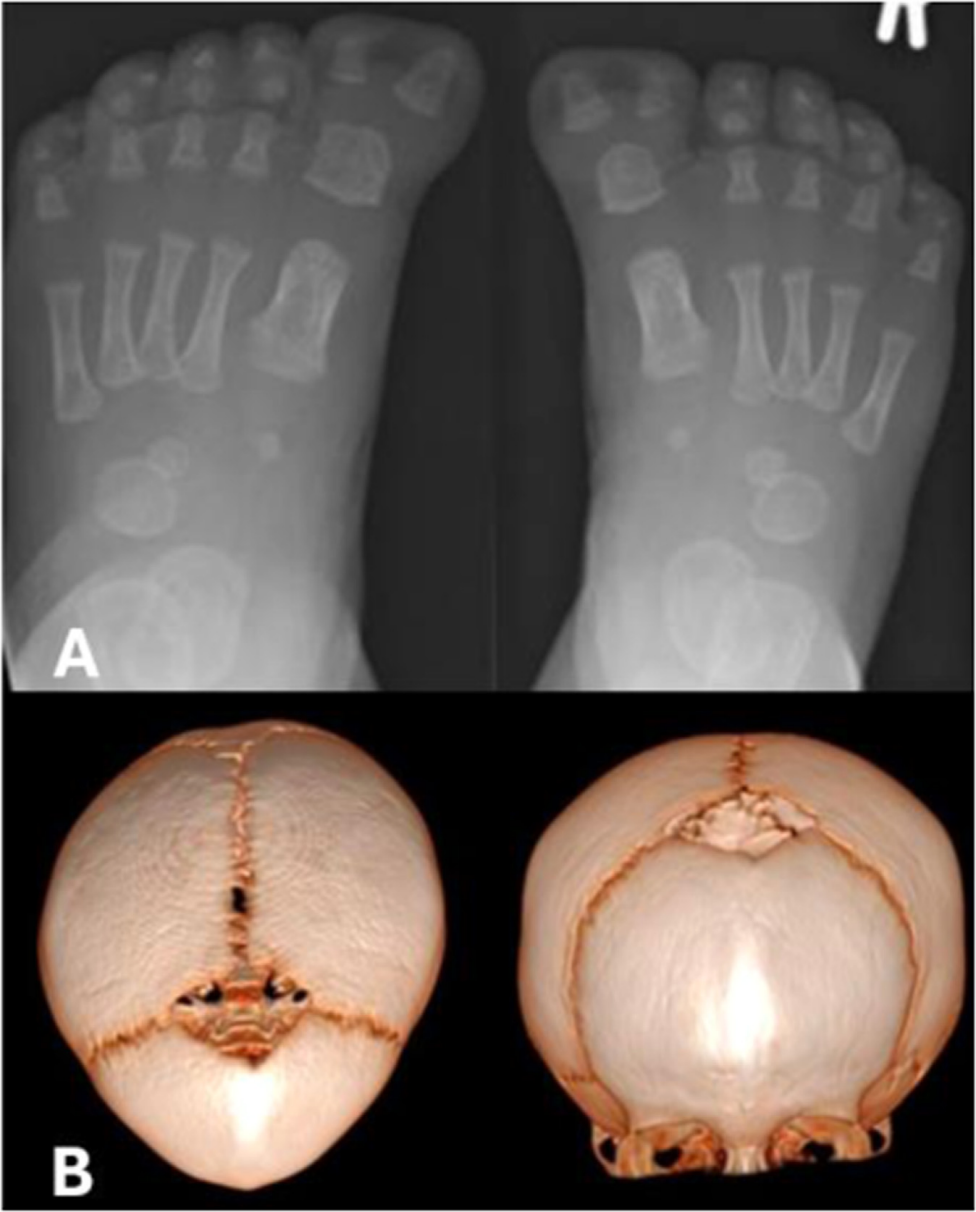
(A) Left and right foot x-rays showing a duplicated distal phalanx of each great toe. (B) 3D reconstruction of computed tomography scan. Trigonocephalic appearance with triangular forehead, absent/fused metopic suture and metopic ridge.

Based on the findings of macrocephaly and polysyndactyly, genetic testing for Greig’s syndrome (cephalopolysyndactyly) was performed, however molecular analysis of *GLI3* was normal. Additionally, on MLPA analysis of *ROR2* and *HOXD13*, no deletion or duplication was detected Figure 2

Trio-based whole exome sequencing was performed. Libraries were constructed using the Illumina DNA Prep with Enrichment kit and capture was performed using Human Core Exome and Human RefSeq Panel probes from Twist Bioscience. Libraries were sequenced on a NovaSeq 6000 instrument using an S2 flow cell (2 × 100 bp) (Illumina). Analysis was carried out applying the DDG2P gene panel (1936 Developmental Disorder Genotype-to-Phenotype genes).^8^ Confirmatory Sanger sequencing was performed. This identified the heterozygous, de novo, NFIA variant c.597dup p.(Ala200ArgfsTer2). The variant was assessed according to ACGS Best Practice Guidelines for Variant Classification in Rare Disease and was classified as pathogenic.^9^ The variant is predicted to result in a frameshift with the transcript likely to undergo nonsense-mediated decay (variant occurs in exon 3 of 11) and has not been reported within the Genome Aggregation Database (gnomAD, v4.1.0). *NFIA* nomenclature is based on NCBI reference sequence NM_001134673.4.

Considering this newly detected *NFIA* variant, a urinary tract ultrasound was performed, showing no abnormalities. Excision of duplicate great toe distal phalanges was performed at 12 months of age. Fronto-orbital advancement and remodelling (FOAR) to correct the trigonocephaly was performed at 21 months 20 days of age.

The child progressed well post-surgery, and at the time of writing, is in early years education receiving specialist input for delayed speech development. Supplementary material (figure 3) shows the child’s progress.

## Discussion

Review of the existing literature consisted of a PubMed search using terms “nfia”, “variant”, “craniosynostosis”, “synostosis” and “nfia” in combination with Boolean logic. No restrictions on date of publication. The database search was complemented with manual review of the reference lists of relevant articles, which resulted in a few additional articles included in the study.

Table 1 details the six documented cases of craniosynostosis associated with an *NFIA*-related disorder.^4^, ^5^, ^6^, ^7^

**Table 1:** Cases of Craniosynostosis associated with *NFIA-*related disorder.

Authors	Case	*NFIA* variant	Phenotypic traits
Rao et al.,^4^	Metopic synostosis in an 8-year-old female	De novo microdeletion of 120 Kilo base pair (Kb) in chromosome 1p31.3 region affecting exons four to nine; an discrete intragenic *NFIA* microdeletion	Macrocephaly, hypoplasia of the corpus callosum, urinary tract abnormality (kinking of the right pelvi-ureteric junction) and developmental delay
Nyboe et al.,^5^	Familial case of affecting four family members, with three out of four having craniosynostosis (two lambdoid, one sagittal).	A 109Kb microdeletion of the gene affecting exons one and two was found in all family members.	Hypoplasia of the corpus callosum, ventriculomegaly, developmental delay and renal tract abnormalities
Tonne et al.,^6^	A 15-year-old boy who presented with metopic craniosynostosis	Two de novo *NFIA* variants found within exon 2 of the *NFIA* gene and resulted in induction of premature stop codons	Thinning of the corpus callosum, macrocephaly and mild developmental delay
Bertini et al.,^7^	A 2-year-old male toddler who presented with sagittal craniosynostosis	A de novo 1.484 Kb *NFIA* gene deletion	Macrocephaly, low-set ears, brachydactyly with bilateral proximally placed first fingers and short lower limbs, speech delay, corpus callosum hypoplasia and dysmorphic lateral ventricles

*NFIA*-related disorder is caused by a heterozygous inactivation or disruption of *NFIA,* without involvement of adjacent or surrounding genes. In addition to isolated *NFIA* disruption, deletion of *NFIA* can also occur as part of a multigenic microdeletion, such as 1p32-p31 deletion syndrome, of which there are less than ten cases reported. However, because *NFIA* is now widely viewed as the critical gene responsible for the 1p32-p31 deletion syndrome phenotypic traits, it is thought to be, itself, an *NFIA*-related disorder. Craniosynostosis has only on two occasions been described as a feature of 1p32-p31 deletion syndrome (with the *NFIA* gene encompassed by the multigenic deletion).^10^

The patient in the present study is unique not only in terms of the genetic variant, but also in terms of his phenotype. The patient exhibits classical features of a *NFIA*-related disorder (macrocephaly, corpus callosum hypoplasia and speech delay) whilst also presenting with preaxial polysyndactyly - only reported once before as a *NFIA*-related disorder and in that case, craniosynostosis was absent.^9^ Similarly, in *NFIA*-related disorder, metopic synostosis has only previously been reported twice.^12^ Overall, given the absence of seizures to date, and normal renal tracts on ultrasound, the newly detected *NFIA* variant c.597dup p.(Ala200fs) would appear to be associated with a milder phenotype of *NFIA*-related disorder. Genetic counselling will be required when this patient reaches young adulthood due to the autosomal dominant inheritance pattern of *NFIA*-related disorders.

## Conclusion

This new *NFIA* variant is important for expanding the knowledge base of this rare condition, and the case strengthens the hypothesis that *NFIA* haploinsufficiency is a cause of craniosynostosis. This is supported by the decision of the NHS Genomic Medicine Service in England to ‘green-list’ the connection between syndromic craniosynostosis and the *NFIA* gene. Genetic testing for *NFIA* may be warranted in craniosynostosis presentations where the routine genetic tests are negative.

## Declaration of competing interest

None of the authors involved in this case report have any competing interests to declare.

## Appendix Supplementary materials

Image, application 1
