# Supplementary material for: Detection of a new heterozygous pathogenic NFIA variant in metopic craniosynostosis with preaxial polysyndactyly: A case report
Source: JPRAS Open. 2025 Jun 3;45:170–5. doi: 10.1016/j.jpra.2025.05.013 (PMC12221604; doi:10.1016/j.jpra.2025.05.013)
Supplement: Supplementary file 1 [file mmc1.pdf]

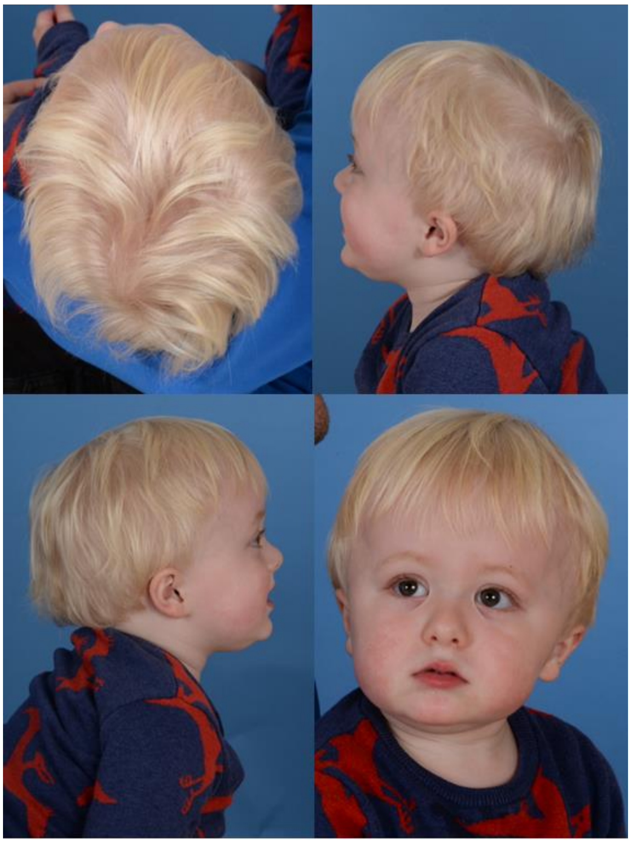

Supplemental Figure 3: Clinical photographs 8 months post-surgery. Corrected trigonocephaly with good forehead symmetry. Macrocephaly persists.
